# Evolutionary adaptation of the Polycomb repressive complex 2

**DOI:** 10.1186/s13072-022-00439-6

**Published:** 2022-02-22

**Authors:** Sabrina Fischer, Lisa Marie Weber, Robert Liefke

**Affiliations:** 1 Institute of Molecular Biology and Tumor Research (IMT), Philipps University of Marburg, 35043 Marburg, Germany; 2 Department of Hematology, Oncology, and Immunology, University Hospital Giessen and Marburg, 35043 Marburg, Germany

**Keywords:** Polycomb, PRC2, Chromatin, Evolution, EPOP, PALI1, JARID2, Drosophila, CpG Islands, EZHIP

## Abstract

The Polycomb repressive complex 2 (PRC2) is an essential chromatin regulatory complex involved in repressing the transcription of diverse developmental genes. PRC2 consists of a core complex; possessing H3K27 methyltransferase activity and various associated factors that are important to modulate its function. During evolution, the composition of PRC2 and the functionality of PRC2 components have changed considerably. Here, we compare the PRC2 complex members of Drosophila and mammals and describe their adaptation to altered biological needs. We also highlight how the PRC2.1 subcomplex has gained multiple novel functions and discuss the implications of these changes for the function of PRC2 in chromatin regulation.

## Background

Cell type-specific gene transcription enables each cell in the body to fulfill its designated function. The correct balance between activating and repressive gene regulatory mechanisms is the foundation for accurate transcription of each single gene [1]. Trithorax group (TrxG) proteins act as transcriptional activators, while Polycomb group (PcG) proteins are key repressive regulators involved in this process [1]. The term “Polycomb” refers to a specific phenotype observed in the model organism *Drosophila melanogaster*, which is characterized by the development of additional sex combs on the second and third pairs of legs [2, 3]. This phenotype was first discovered in 1947 after the mutation of a specific gene [4], then named Polycomb (Pc), but later observed upon mutation of further genes, now collectively referred to as Polycomb group (PcG) genes [3]. These mutations lead to the misexpression of homeotic genes that are vital for initiation of the correct developmental program [5]. Subsequent characterization of the PcG genes and their protein products demonstrated that these proteins participate in gene repression [2, 3]. The importance of the PcG genes during developmental processes has been confirmed in mice and other organisms, where their deletion often leads to embryonic lethality [6].

Biochemical characterization in Drosophila and mammals [7, 8] revealed that the Polycomb proteins can assemble into several distinct protein complexes, with the Polycomb repressive complex 1 (PRC1) and -2 (PRC2) being the most important [6]. These two complexes work synergistically to establish a repressive chromatin state at their target genes [9]. The PRC1 complex facilitates ubiquitination at H2AK119 [10], which is essential for the interplay of the distinct Polycomb complexes [11–13]. The dominant function of PRC2 is to deposit the H3K27me3 histone mark [8, 14], which is a common feature of repressive chromatin [15]. In more distant organisms the Polycomb complexes have a comparable role, but they differ greatly in their composition and functionality. Several excellent reviews cover Polycomb complexes in plants and *C.elegans* [16–18].

Although the general repressive role of PRC2 is retained during evolution, its functionality has diversified substantially. In this review, we will address the distinct composition and function of PRC2, with a focus on PRC2 in Drosophila and humans. Here we discuss the relevance of novel functions of mammalian PRC2, with particular emphasis on the PRC2.1 subcomplex. We also consider why this subcomplex appears to have achieved greater diversity compared to PRC2.2.

## Main text

### PRC2 complex composition in Drosophila and mammals

**Fig. 1:**
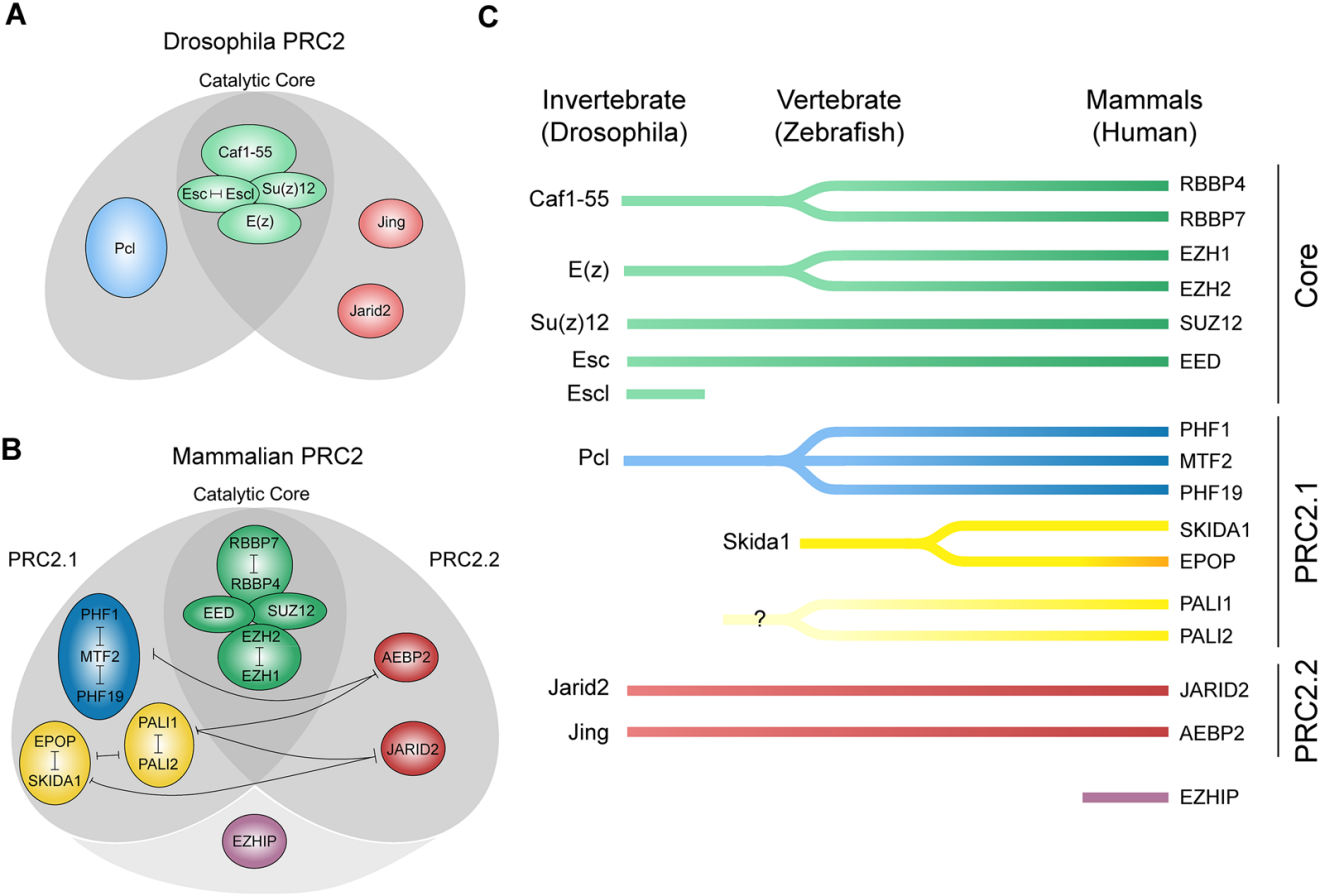
Overview of the main PRC2 members and their competition in Drosophila (**A**) and mammals (**B**), as well as their evolutionary occurrences (**C**). Mammalian complexes with one of the Polycomb-like proteins (PHF1, MTF2, or PHF19) are named PRC2.1, while complexes including AEBP2 and JARID2 are referred to as PRC2.2. Related complex members in Drosophila and mammals are indicated by similarly colored groups. Members of the catalytic core are illustrated in green, whereas Pcl and the respective PRC2.1 subcomplex members are indicated in blue. Mammalian PRC2.1 can have additional proteins such as EPOP, SKIDA1, and PALI1/2 (yellow), which compete against each other, allowing multiple PRC2.1 complex compositions (see also Fig. 6). Jing, Jarid2, and the respective mammalian PRC2.2 subcomplex members AEBP2 and JARID2 are colored red. The mammalian-specific protein EZHIP (purple) interacts with PRC2 in a subcomplex-independent manner

The early characterization of PRC2 in Drosophila has demonstrated that PRC2 is composed of a core (Fig. 1A), consisting of the histone methyltransferase E(z) and the proteins Su(z)12, Esc/Escl, and Caf1-55 [8]. The general composition of the PRC2 core is basically identical in mammals [7], with two paralogs of the methyltransferase, EZH1 and EZH2, and the proteins SUZ12, EED and either RBBP4 or RBBP7 (Fig. 1B). This catalytic core is sufficient to methylate histones in vitro [7, 8]. However, the PRC2 core does not act alone but in concert with additional proteins, which extend and customize the activity of the core complex. In Drosophila, the Polycomb-like protein (Pcl) is essential for efficient H3K27 methylation [19]. The core can further be associated with Jarid2 (also named Jumonji) and Jing [3]. In mammals, several independent studies [20–25] indicated that PRC2 typically contains either one of the Pcl homologs (PHF1, MTF2, PHF19), or the homologs of Jarid2 and Jing, JARID2 and AEBP2, demonstrating the establishment of two major PRC2 subcomplexes, commonly referred to as PRC2.1 and PRC2.2, respectively (Fig. 1B). Although it has not yet been conclusively confirmed that Drosophila PRC2 forms similarly distinct subcomplexes, it is likely that the PRC2 core preferentially interacts with either Pcl or Jarid2, which may explain their nonredundant functions [19, 26].

Recent work demonstrated that mammalian PRC2.1 not only contains a Pcl homolog but can also be associated with additional proteins, for which no homologs are known in Drosophila (Fig. 1B, C). At least three proteins are associated mainly with the PRC2.1 subcomplex: EPOP, PALI1, and PALI2 [21, 22, 27] (Fig. 1B). The protein SKIDA1 (C10orf140), which shares the PRC2-binding domain with EPOP [28], was also found to be associated with PRC2 [20, 29] and is likely to associate specifically with PRC2.1 (Fig. 1B). PRC2.1 contains at least one of two methyltransferases (EZH1 or EZH2), and one of the three Polycomb-like proteins (PHF1, MTF2, or PHF19), resulting in already six possible combinations. It has also been shown that in PRC2.1, EPOP and PALI1 compete for binding [20, 21, 30]. The additional presence of one of either EPOP, SKIDA1, PALI1, or PALI2 therefore generates twenty-four further putative combinations. These are all referred to as “PRC2.1”. Thus, the term “PRC2.1” refers to a large family of similar, yet distinct, complexes in which the PCL proteins are common to all members, in combination with a wide range of signature subunits. Notably, in the absence of AEBP2, JARID2 and MTF2 were found to be present within the same complex [23]. This suggests that the distinction between PRC2.1 and PRC2.2 is not strict, and that hybrid PRC2 complexes can form when members of PRC2.1 and PRC2.2 target nonoverlapping interaction sites [31]. In addition to the proteins that preferentially are either part of PRC2.1 or PRC2.2, the PRC2-associated protein EZHIP directly interacts with EZH2 and influences the function of PRC2 in a subcomplex-independent manner [32–35] (Fig. 1B).

In mouse embryonic stem cells (mESCs) the members of PRC2.1 and PRC2.2 colocalize at the majority of PRC2 binding sites [36, 37]. In these cells, only the simultaneous removal of the associated proteins of both PRC2.1 and PRC2.2 completely abrogates PRC2 core chromatin recruitment, while single deletions merely lead to reduced recruitment [36, 37]. These results demonstrate that the efficient recruitment of PRC2 requires the associated factors of both PRC2 subcomplexes, suggesting a certain redundancy and synergism between PRC2.1 and PRC2.2. Nonetheless, recent work demonstrated that PRC2.1- and PRC2.2-associated factors have unique roles in chromatin binding and mouse ES cell differentiation [38–41]. In mESCs, PRC2.1 chromatin recruitment is mainly mediated by the DNA binding function of MTF2, while PRC2.2 chromatin recruitment is mediated by the affinity of JARID2 for the PRC1-associated histone mark H2AK119ub [38]. These alternative binding modes may explain why shifting the balance of PRC2 towards PRC2.1 using specific point mutations within SUZ12 leads to a strongly increased affinity of PRC2 for chromatin [40]. Consistent with this hypothesis, the removal of MTF2 or JARID2 in mESCs leads to the derepression of a distinct set of genes upon differentiation and consequently to altered lineage choices. MTF2 is associated with genes that remain repressed during differentiation [39] and its loss leads to enhanced commitment to all germ layers [41]. Conversely, JARID2-sensitive genes were active in mESCs and became newly repressed upon differentiation [39]. JARID2-deleted cells possess a more selective differentiation process towards early differentiated precursors and have a reduced efficiency towards mesendodermal lineages [41]. Thus, this recent work suggests that each of the PRC2 subcomplexes has a unique function, which cannot be reproduced by alternative complex assemblies. Further work is therefore required to unravel the specific functions of the PRC2-associated factors.

### Evolution of the PRC2 core

**Fig. 2:**
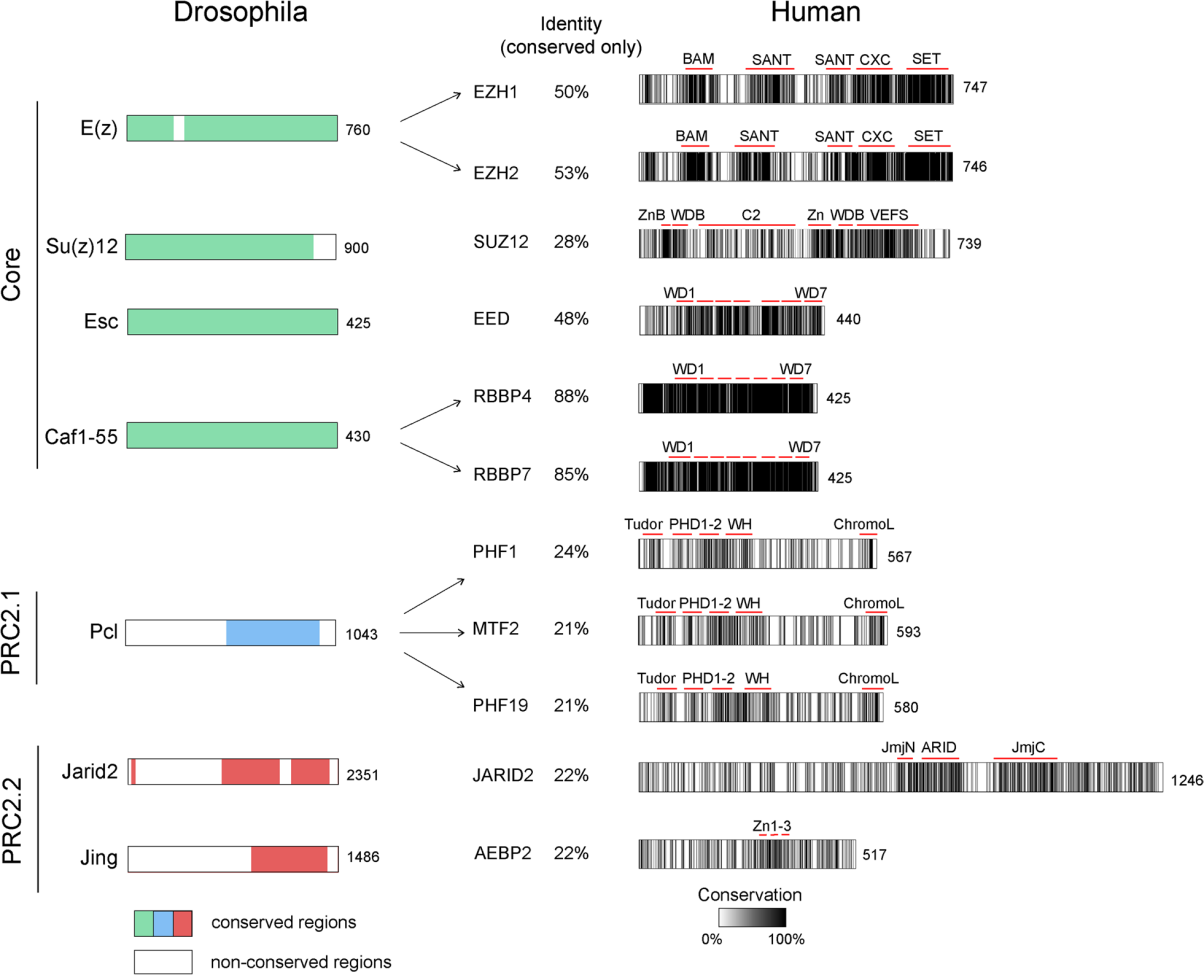
Comparison of PRC2 members that exist in both Drosophila and humans. On the left side, the regions of the Drosophila proteins that are also present in human proteins are colored. On the right side, the identity and conservation of the retained regions are shown. Annotated domains are highlighted. BAM: β-addition motif; SANT: SANT (Swi3, Ada2, N-CoR, and TFIIIB) domain; CXC: cysteine-rich domain; SET: SET (Su(var)3–9, Enhancer-of-zeste and Trithorax) domain; WDB: WD40-repeat binding; ZnB: zinc finger binding helix; C2: C2 domain; Zn: zinc finger; VEFS: VEFS (VRN2-EMF2-FIS2-Su(z)12)-Box; WD: W40 repeat; PHD: plant homology domain; WH: winged-helix domain; JmjN: Jumonji N domain; JmjC: Jumonji C domain; ARID: A–T rich interaction domain; ChromoL: chromo-like domain

As described above, some but not all components of PRC2 are represented in both Drosophila and mammals (Fig. 1). As expected, the proteins that make up the PRC2 catalytic core are generally well conserved during evolution. The structural proteins RBBP4/7 are more than 80% identical to the Drosophila homolog Caf1-55, suggesting that these proteins have evolved little from the common ancestor protein (Fig. 2). In contrast, the other core proteins show a lower sequence identity of approximately 50% for EZH1/2 and EED and less than 30% for SUZ12. This relatively low sequence identity demonstrates that these genes have considerably diverged during evolution. Most pronounced is the development of the methyltransferase *E(z)*. The ancestral *E(z)* gene was duplicated during vertebrate evolution [42], leading to two genes in mammals, *EZH1* and *EZH2* (Fig. 1C). Both mammalian enzymes have retained H3K27me3 methylation activity [43], but the protein sequence of EZH2 is more closely related to the Drosophila homolog E(z). A large body of work suggests that EZH2 is the main PRC2 methyltransferase in mammals and is responsible for the deposition of most H3K27me3 marks [43, 44]. Consistently, EZH2 knockout leads to a strong reduction in H3K27me3 and is embryonically lethal [6, 43]. The absence of EZH2 also leads to a reduction in H3K27me2 and H3K27me1, but to a lesser extent than the reduction of H3K27me3, suggesting that the remaining EZH1 can compensate for the loss of EZH2 and can deposit H3K27me1 and H3K27me2 [44]. Nonetheless, EZH1 is generally considered less relevant for the function of PRC2 since it is not embryonically lethal [6, 42]. The establishment of two distinct methyltransferases within PRC2 indicates a special EZH1-related function of PRC2. Interestingly, biochemical studies suggest that PRC2 containing EZH1 can compact chromatin in a methyltransferase-independent manner [43, 45]. Additionally, recent structural work demonstrated that the EZH1-PRC2 has a substantially different conformation than EZH2-PRC2 and that the previously observed chromatin compaction is mainly facilitated by EZH1-PRC2 dimers [46]. Although EZH2-PRC2 can also establish dimeric states, this conformation appears to have other functions, such as enhanced chromatin and RNA binding [25, 47]. Thus, these preliminary studies suggest that EZH1-containing PRC2 may have functions different from EZH2-containing PRC2, which might explain, for example, the EZH1-specific biological role in hematopoiesis [48, 49]. More work will be required to better understand the mechanistic and biological function of EZH1-containing PRC2.

In addition to E(z), also the Su(z)12/SUZ12 proteins has diverged during evolution. Although the general domain structure of SUZ12 is conserved, the sequences of Drosophila and human proteins display only 30% identity (Fig. 2). Considering that the SUZ12 protein acts as a binding hub, required for the assembly of PRC2 [31], one could speculate that these rather substantial differences in the primary sequence of SUZ12 compared to Su(z)12 are possibly necessary to allow the binding of additional proteins unique to mammals, such as EPOP and PALI1. This idea is supported by recent structural work that demonstrated that the less-well conserved N-terminal part of SUZ12 (Fig. 2) acts as a binding platform for several associated proteins such as AEBP2, JARID2, PHF19, and EPOP [31].

In contrast to Su(z)12/SUZ12 and E(z), the changes to the core member Esc/EED are less pronounced. Drosophila Esc and the human EED proteins are more than 50% identical. Esc/EED consists of seven WD40 domains that form a beta propeller and function as a critical structural component of PRC2 [50]. Esc/EED deletion almost completely abrogates the activity of PRC2 in Drosophila [51] and mammals [52, 53], demonstrating the importance of this protein. Esc/EED interacts with the H3K27me3 mark [54], which is required for steric activation of PRC2 [50, 55] and thus the propagation of the repressive histone mark. This critical function is facilitated by the highly similar WD domains of Esc/EED (Fig. 2) and is thus conserved between Drosophila and mammals [54].

Notably, in Drosophila, a homolog of Esc exists, called Esc-like (Escl), which mainly differs from Esc at the N-terminus [56, 57]. It shows a distinct expression pattern, and it has been shown that PRC2 with Escl has reduced methyltransferase activity [56, 57]. In mammals, only one EED homolog exists, which, however, produces four alternative isoforms with varying N-termini [58]. PRC2 with an N-terminally truncated EED has reduced methyltransferase activity towards the noncanonical substrate H1K26, at least in vitro [58]. Thus, this work implies that alternative Esc/EED proteins may be involved in modulating the methyltransferase activity of PRC2. However, the biological relevance and molecular differences of the distinct Esc/EED proteins remain uncertain.

Importantly, PRC2 is not only allosterically activated upon binding of Esc/EED to the H3K27me3 mark, but also inhibited by active chromatin marks, such as H3K4me3, H3K36me2, and H3K36me3 [59]. In contrast to the in *trans* acting H3K27me3 [50], the inhibition by the active histone marks is established in *cis*, meaning that active histone marks on a specific histone tail inhibit PRC2 from depositing the H3K27me3 mark on the very same tail [59–61]. Mechanistically, it has been shown that the H3K36me3 mark occupies a critical position at the PRC2 nucleosome interaction interface, therefore directly influencing the activity of PRC2 [62]. Similar to the allosteric activation by H3K27me3, this inhibitory function of active histone marks is evolutionarily conserved [62].

Collectively, during evolution the PRC2 core has largely retained the ability to form a complex and facilitate H3K27me3 deposition. Both allosteric activation by H3K27me3 and inhibition by active histone marks are conserved between Drosophila and mammals.

### Evolution of the PRC2.1 subcomplex

#### The Polycomb-like proteins (PCLs)

The Polycomb-like (Pcl) protein was first described in the Drosophila system to participate in a Polycomb-related function [63]. Further work confirmed that Pcl and its mammalian homologs are components of PRC2 [64, 65] and are required to generate high levels of H3K27me3 [19, 65]. In Drosophila, Pcl is part of a subset of PRC2 that does not contain Jarid2 [19], which is in line with the results from mammalian PRC2, where homologous Polycomb-like proteins (PHF1, MTF2, PHF19; also PCL1, PCL2, PCL3) have been linked to the PRC2.1 subcomplex [20]. Accordingly, the phenotype of mutants or knockout of Pcl or the respective mammalian homologs is weaker compared to PRC2 core proteins [19, 66, 67]. However, numerous studies have demonstrated the importance of all three mammalian PCL proteins in physiological and pathophysiological processes, such as DNA repair [68, 69] and cancer cell proliferation [70–73], supporting that they are essential regulators of the PRC2 function.

Structurally, the PCL proteins possess five globular domains, namely a Tudor domain, two PHD fingers, a winged-helix domain, and a C-terminal chromo-like domain. This general structural composition is conserved between the Drosophila Pcl and its vertebrate paralogs PHF1, MTF2 and PHF19. The C-terminal chromo-like domain was identified as necessary and sufficient for the interaction with PRC2 [31, 74, 75]. Recent work suggests that this domain interacts with the N-terminal region of the PRC2 core component SUZ12 [31] and is involved in establishing a dimeric state of mammalian PRC2, which in turn increases the binding to chromatin [25]. Considering that PRC2 dimers have also been observed in Drosophila [47, 64], it is likely that this function of the chromo-like domain is conserved.

In contrast to the chromo-like domain, the function of some of the other domains has diverged during evolution. Through an aromatic cage, the Tudor domain of all three mammalian paralogs can recognize the H3K36me3 histone mark [69, 74, 76–79]. This aromatic cage cannot be formed in the Drosophila ortholog due to the absence of two aromatic amino acids [80]. Consequently, Pcl is not able to bind to H3K36me3, supporting the view that this function is not required in Drosophila [80]. H3K36me3 binding is similar in all three mammalian paralogs [76], indicating an important biological function. It has been proposed that the H3K36me3 binding of mammalian PCLs is involved in the recruitment of PRC2 to chromatin [74]. However, no obvious overlap between Polycomb-like proteins and H3K36me3 on chromatin has been observed so far [74]. Furthermore, H3K36me3 has been demonstrated to inhibit the enzymatic activity of PRC2, independent of the PCL proteins [59, 62]. The potential interplay between this repressive function of H3K36me3 towards PRC2 and the H3K36me3 binding of the PCL proteins awaits further clarification. One possibility is that the Tudor domains not only bind to H3K36me3 but also influence the DNA accessibility. At least in the case of PHF1, it has been observed that the region N-terminal to the Tudor domain increases DNA accessibility, which may be important to increase the binding of PRC2 to chromatin [77, 78]. Interestingly, in vitro studies suggest that the Tudor domains of PHF1 and PHF19 also have a weak affinity for trimethylated lysine 27 of the specific histone variant H3t [81, 82]. This histone variant differs from canonical histone H3 by four amino acids, and is highly expressed in the testis, but can also be found in other organs, such as the brain [81]. Thus, one could speculate that the Tudor domains of the PCLs could be essential for PRC2 recruitment in H3t-expressing tissues.

In addition to the Tudor domain, the function of the winged-helix domain has diverged during evolution. In mammals, this domain recognizes unmethylated CpG motifs, which are commonly present in CpG islands (CGIs) [67, 83]. This ability may contribute to the commonly observed recruitment of PRC2 to CGIs in many cell types [84]. A sequence of four positively charged amino acids (RRKK) is crucial for the recognition of the CpG motif. In Drosophila, where the DNA is mostly unmethylated [85], this sequence is not conserved, explaining why for the Drosophila Pcl homolog only nonspecific DNA binding was observed [75]. In mESCs the PCL protein with the most significant role in the recruitment of PRC2 to CGIs is MTF2, while the other PCL proteins are less relevant for this aspect [36–38]. Consequently, deletion of MTF2 in mouse ES cells strongly reduces the levels of PRC2 at Polycomb target genes [36, 37, 67, 83]. Residual PRC2 recruitment after MTF2 deletion is likely facilitated by the other Pcl homologs or Jarid2 [36, 37].

In addition to changes in the domain function, other novel features have been acquired by the PCL proteins during evolution. Specifically, in PHF1 the N-terminal PHD finger has adapted to enable an interaction with the tumor suppressor p53 [70, 71]. Two for the interaction essential serine residues are present in PHF1, but not in PHF19 or MTF2 or in the Drosophila homolog Pcl [70]. This p53 binding function is important for the promotion of cellular quiescence and may explain why this gene is often downregulated in cancer [27, 70]. It is possible that other nonhomologous regions of PHF1, MTF2, or PHF19 may possess isoform-specific functions that have not yet been revealed. Further interacting partners, such as the PHF19-interacting histone demethylase RIOX1 (NO66) [86], may also bind to isoform-specific sequences.

#### EPOP and SKIDA1

EPOP (Elongin BC and Polycomb repressive complex 2-associated protein) is a mammalian-specific PRC2-associated factor [22, 27, 30]. Thus, EPOP belongs to the evolutionarily youngest PRC2 components and is mostly unstructured [28]. A conserved C-terminal region is responsible for the interaction with the PRC2 core member SUZ12 [31]. EPOP enhances the methyltransferase activity of PRC2 in vitro [22, 87], but the exact mechanisms of this influence remain to be determined.

EPOP and JARID2 are not commonly found in the same complex [20, 22, 27, 30], suggesting competition in the interaction with PRC2. In line with these observations, EPOP deletion increases JARID2 chromatin binding [22] and leads to an increased chromatin association of PRC2 and enhanced repression of PRC2 target genes [22, 27]. This finding suggests that EPOP counteracts PRC2 in vivo, in contrast to its in vitro role, implicating a rather complex functional role of EPOP.

Interestingly, EPOP interacts not only with PRC2 but also with the heterodimer Elongin BC [22, 27], which is involved in transcription elongation and protein turnover [88]. Elongin BC interacts with EPOP via a BC-box at the N-terminus of EPOP [27]. Experiments in mouse ES cells suggest that EPOP works together with Elongin BC to elevate gene transcription at actively transcribed genes, which may also involve the deubiquitinase USP7 [27]. EPOP, Elongin B, and Elongin C are often upregulated in cancer [27] and systematic CRISPR screening experiments demonstrated that they are “common essential” in human cancer cell lines [89], meaning that they are required for efficient proliferation in most human cancer cells. However, the function of Elongin BC towards EPOP and PRC2 as well as the reasons why EPOP and Elongin BC are required for cancer cell growth are currently unknown.

**Fig. 3:**
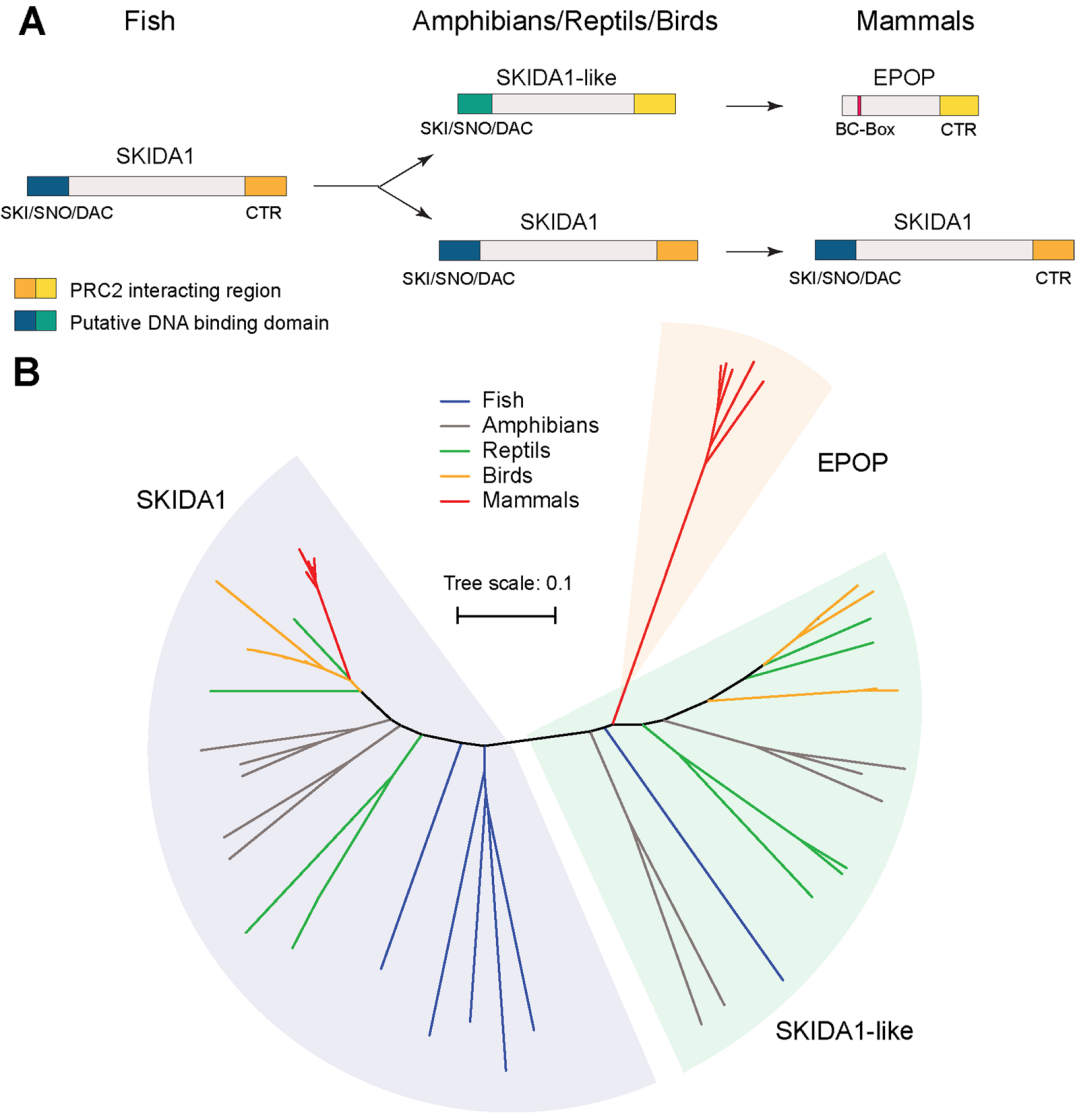
Schematic overview of the evolution of the SKIDA1, SKIDA1-like, and EPOP proteins. **A** The C-terminal region (CTR) of the proteins binds to the PRC2 core [31] and is conserved among all proteins. In SKIDA1 and SKIDA1-like the N-terminal region contains a putative DNA binding SKI/SNO/DAC domain, which is replaced by a BC-box-containing region in EPOP. **B** Phylogenetic comparison of SKIDA1, SKIDA1-like and EPOP proteins from five representative organisms in fish (*Astyanax mexicanus*, *Esox lucius*, *Fundulus heteroclitus*, *Danio rerio*, *Lepisosteus oculatus*), amphibians (*Xenopus laevis*, *Bufo bufo*, *Microcaecilia unicolor*, *Rhinatrema bivittatum*, *Geotrypetes seraphini*), reptiles (*Alligator mississippiensis*, *Anolis carolinensis*, *Chrysemys picta bellii*, *Protobothrops mucrosquamatus*, *Thamnophis sirtalis*), birds (*Coturnix japonica*, *Apteryx australis mantelli*, *Haliaeetus leucocephalus*, *Pseudopodoces humilis*, *Parus majo*) and mammals (*Homo sapiens*, *Balaenoptera musculus*, *Equus przewalskii*, *Mesocricetus auratus*, *Capra hircus*), respectively, using ClustalOmega [91]. The tree was visualized using iTOL [92]

The C-terminal PRC2-interacting region of EPOP is 52% homologous to the C-terminus of another relatively uncharacterized protein, named SKIDA1 (C10orf140) [28]. SKIDA1 has been identified by IP-MS (immunoprecipitation followed by mass spectrometry) experiments using PRC2 components as bait, which suggests that it can also interact with PRC2 [20, 29]. Given the relatively high homology of the PRC2 interacting region [28], SKIDA1 is likely equally associated with PRC2 as EPOP and may also favor the formation of PRC2.1. The N-terminal part of SKIDA1 contains a SKI/SNO/DAC domain (Fig. 3), which is a putative DNA binding domain, potentially allowing the recruitment of PRC2 to specific genomic sites. To date, the biological role of SKIDA1 and its role in PRC2 have not been investigated. Interestingly, however, the SKIDA1 gene has been demonstrated to be regulated by the Polycomb system itself [90], suggesting a potential feedback mechanism.

Investigation of the evolutionary development of EPOP and SKIDA1 using data from NCBI and Ensembl suggests that the mammalian *SKIDA1* and *EPOP* genes were likely derived from the same ancestral gene, which first emerged in early vertebrates. Later, the *SKIDA1* gene appears to have undergone a gene duplication producing a closely related *SKIDA1*-like gene (Fig. 3). Both, *SKIDA1* and the *SKIDA1*-like genes are found in the genomes of some fish and in most amphibia, reptiles, and birds. Importantly, in mammals, while the *SKIDA1*-like gene does not exist, the *EPOP* gene can be found. EPOP shares only the conserved C-terminal region with the SKIDA1-like protein, while the other parts of the proteins show no homology. It is possible that the N-terminal part of the *SKIDA1*-like gene has been replaced by a new sequence of unknown origin, forming the new *EPOP* gene. Notably, EPOP’s BC-box is a mammalian-specific feature, making it likely that the link between PRC2 and Elongin BC is restricted to mammals [22, 27] (Fig. 3).

#### PALI1 and PALI2

**Fig. 4:**
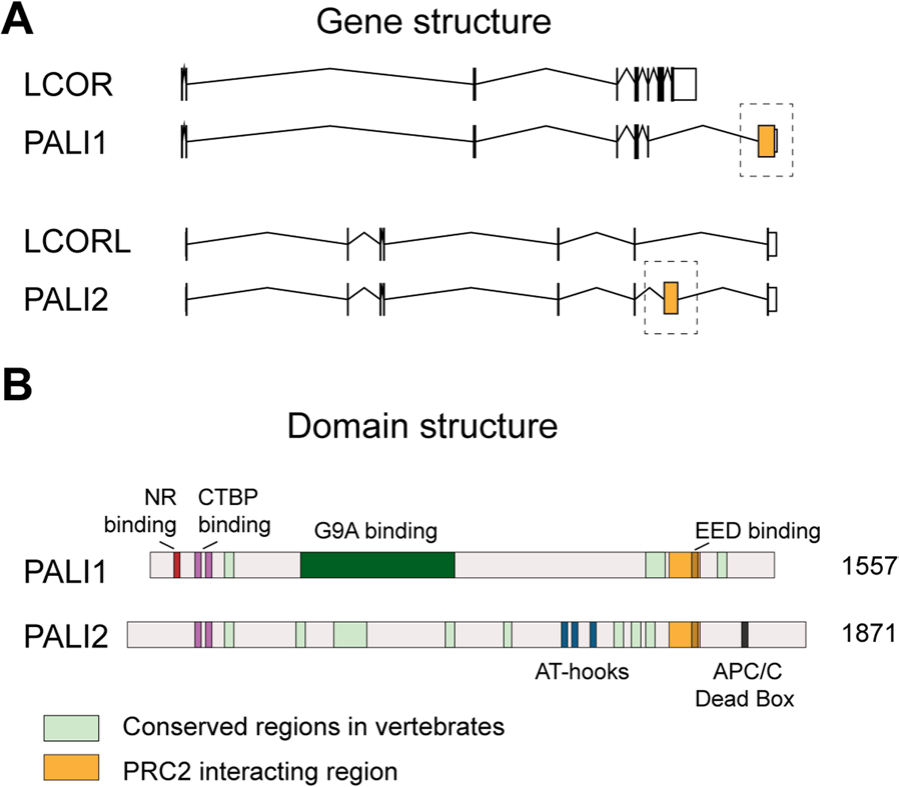
Gene and domain structures of *PALI1* and *PALI2*. **A** *PALI1* and *PALI2* are splicing variants from the *LCOR* and *LCORL* genes, respectively, with C-terminally located PRC2 interacting regions. **B** Both PALI1 and PALI2 interact with CTBP proteins via a homologous N-terminal domain. While PALI1 also interacts with the G9A complex and possesses an N-terminal nuclear receptor (NR) binding motif, PALI2 has three AT hooks, putatively interacting with DNA

PALI1 (PRC2 associated LCOR isoform 1) is another recently described PRC2-interacting protein found only in vertebrates [21]. It is produced from a specific splice isoform of the *LCOR1* gene that introduces an exceptionally large exon (previously annotated as C10orf12), increasing the size of the LCOR1 protein by a factor of three (Fig. 4). Only the region present in PALI1, but absent from LCOR1, contains a PRC2 interacting domain. In IP-MS analysis, PALI1 was found to be associated with the PRC2 core members and PCL proteins, suggesting that PALI1 belongs to the PRC2.1 subcomplex [21]. Because PALI1 was not found together with EPOP [20, 21, 30] and vice versa [22, 27], it was suggested that PALI1 and EPOP interact with a similar region of the PRC2 core and therefore compete for binding (Fig. 1). Potentially, PALI1 shifts the composition of PRC2 towards PRC2.1, similar to EPOP [22]. Another model presents PALI1 as a functional antagonist of AEBP2, central to balancing the activities of PRC2.1 and PRC2.2 [21].

Recent work suggests that PALI1 can allosterically activate PRC2 [93], comparable to JARID2 [94, 95]. Mechanistically, PRC2 methylates PALI1 at a sequence that is closely related to a sequence found in JARID2. When methylated, PALI1 can bind to EED, leading to the allosteric activation of PRC2 [93]. This allosteric activation likely explains why PALI1 promotes the methyltransferase activity of PRC2 and why the knockout of PALI1 leads to reduced levels of H3K27me3 in mouse embryos [21, 93].

PALI1 not only interacts with PRC2 but also with many additional chromatin regulators such as CTBP1 and the G9A (EHMT2) complex, which are involved in gene repression [21]. At some PRC2 targets, the recruitment of PRC2 depends on G9A complex activity [96], possibly due to the action of PALI1. Additionally, PALI1 possesses a nuclear receptor binding LXXLL motif, which may implicate PALI1 in nuclear receptor regulation [21]. These findings suggest that PALI1 bridges different protein complexes to act as a versatile chromatin regulator [21]. The importance of PALI1 is indicated by the impaired mouse development seen in PALI1 knockout [21].

PALI1 has a homolog, named PALI2, which, like PALI1, is derived from a specific splice-variant, in this case from the LCORL gene (Fig. 4). PALI1 and PALI2 both possess the PRC2-interaction domain and the CTBP binding motifs. In PALI2, the EED-binding motif important to allosterically activate PRC2 is also present, suggesting that PALI2 may facilitate PRC2 regulation similar to PALI1 [93]. Apart from these features, PALI1 and PALI2 show no extensive homology. In contrast to PALI1, PALI2 possesses three AT-hooks, which may enhance chromatin binding of a PALI2-containing PRC2 [21] (Fig. 4). Currently, no functional data about the role of PALI2 have been obtained. Both PALI1 and PALI2 exist in early vertebrates, such as zebrafish, and are likely derived from a common ancestral gene, related to the Drosophila LCOR homolog *Eip93F* [21]. However, homologs of the PALI1/2-splice isoforms can only be found in vertebrates, suggesting that these proteins have evolved to modulate PRC2 activity in vertebrate-specific pathways [21].

### Evolution of the PRC2.2 subcomplex

#### JARID2

JARID2 was originally identified as “Jumonji” in mice as a transcriptional repressor and is important for correct development in mice [97]. The role of Jarid2/Jumonji was also confirmed in Drosophila [98]. Jarid2 was not identified as a Polycomb protein during initial screens [99], but was later identified as a PRC2 interaction partner [24, 100–103]. “Jumonji” was eponymous for the so-called Jumonji family of proteins [97] that have α-ketoglutarate-dependent histone demethylase activity [104]. JARID2 itself has no known demethylase activity because the catalytic center of JARID2’s Jumonji domain is not configured for substrate binding [100]. Currently, the function of the Jumonji domain of JARID2 remains unknown [100].

JARID2 interacts with PRC2 via a sequence in the N-terminal part of the protein [101], which associates with SUZ12 and EED [95] and is essential for the ability of JARID2 to repress transcription [101]. To fulfill its function with respect to PRC2, at least two additional features are important. First, JARID2 possesses a ubiquitin-binding sequence that increases the affinity for PRC1-mediated H2AK119-ubiquitinated chromatin [12, 105, 106]. Second, JARID2 is methylated by PRC2 at a conserved N-terminal region (K116 in humans, K46 in Drosophila) (Fig. 2) [94]. The methylated site mimics methylated histone H3 at lysine 27, is bound by EED and increases the catalytic activity of PRC2 by inducing allosteric activation [94, 95]. JARID2, therefore, increases the affinity of PRC2 for chromatin and enhances its methyltransferase activity. Although JARID2 has an nonspecific DNA binding activity due to its ARID domain, this seems to be of minor importance for the function of JARID2 [100]. Notably, most known mechanistic functions of JARID2, including H2Aub binding and allosteric activation [11, 94], are similar between Drosophila and humans, suggesting that the main tasks of JARID2 are conserved.

JARID2 deletion is lethal in mice but phenotypes of different severities appear to depend on the targeting strategy and genetic background [107]. The strongest effects were observed in the neural and cardiac systems [107, 108] and intellectual disability in some human patients has been linked to microdeletions that include *JARID2* [109]. Additionally, in Drosophila, the function of Jarid2 is mainly attributed to neural-related pathways [97], supporting the idea that JARID2, as a component of the PRC2.2 complex, has increased significance in neural lineages.

#### AEBP2

The other main component of the PRC2.2 subcomplex, AEBP2 (in Drosophila annotated as Jing), is a zinc-finger protein originally described as a transcriptional repressor in mice [110] and is involved in brain and wing development in Drosophila [111, 112]. Later research showed that it is associated with PRC2, as demonstrated by mass spectrometry of purified PRC2 from human HeLa cells [14]. Although AEBP2 is not essential for the methyltransferase activity of PRC2, it is still required for its optimal enzymatic activity in vitro [113], likely due to its stabilizing function [95, 106, 114].

Structural studies have shown that AEBP2 mimics an unmethylated H3 tail interacting with the RBBP4 subunit and is thereby important for the stability of PRC2 [95]. The relevant protein sequences are highly similar between Drosophila and humans, suggesting that this function is evolutionarily conserved. In mice, AEBP2 knockout leads to developmental defects and is embryonic lethal [23, 115]. Unexpectedly, the observed phenotype resembles the deletion of Trithorax proteins [23]. This has been explained by the formation of a hybrid PRC2 that consists of members of both PRC2.1 and PRC2.2. This hybrid complex may have a stronger activity than the PRC2.2 subcomplex, leading to a repression of developmental genes and consequently to a Trithorax-related phenotype [23]. Thus, although AEBP2 stimulats the histone methyltransferase activity of PRC2.2 in vitro [113], the in vivo function of AEBP2 is currently less clear.

Taken together, based on the current understanding, both AEBP2 and JARID2 have mostly retained their function upon evolution. The Drosophila and mammalian proteins fulfill their role in PRC2 stabilization, allosteric activation, and H2Aub binding. However, given the change in their primary sequence (Fig. 2), it is likely that mammalian-specific functions of PRC2.2 exist.

### EZHIP—a mammalian specific and subcomplex-independent PRC2 interactor

EZHIP (Enhancer of Zeste Homologs Inhibitory Protein, also known as CATACOMB and CXorf67) is the most recently identified interactor of mammalian PRC2 [32, 116]. It associates with both PRC2.1 and PRC2.2 as shown by IP-MS experiments with EZHIP as bait [32]. The *EZHIP* gene becomes active following DNA demethylation in primordial germ cells [32] and cancer cells [33]. In normal tissues, its expression is highest in the initial stages of development and can be detected in ovaries and testes, but its expression is low in most differentiated tissues [32].

The deletion of EZHIP leads to an increase in H3K27me2/3, while its artificial overexpression leads to a reduction in H3K27me2/3 marks [32, 33, 116]. EZHIP controls H3K27me3 deposition through direct interference with EZH2 activity [116, 117]. The highly conserved C-terminal sequence of EZHIP binds directly to EZH2 and inhibits its activity, which is comparable to the effects of the H3K27M mutation [33–35, 117]. Notably, the EZHIP binding sequence is a more potent inhibitor of PRC2 activity (IC50 = 4.1 µM) than the H3K27M peptide (IC50 = 27.87 µM), likely because the EZHIP peptide reflects the calculated “optimal” substrate sequence previously defined for PRC2 [34, 118]. EZHIP has only a subtle impact on the chromatin binding of PRC2 [32]. However, EZHIP’s inhibitory activity towards PRC2 is particularly efficient when PRC2 is allosterically activated by H3K27me3, leading to a loss of PRC2 spreading and an eventual recession of H3K27me3 back to sites of PRC2 recruitment [35]. Evolutionarily, EZHIP homologs are exclusively found in placental mammals and show little overall sequence conservation except for the PRC2 binding sequence [32, 35], suggesting that the main function of EZHIP is the inhibition of PRC2 during early embryogenesis and in germ cells.

The biological function of EZHIP is just starting to be revealed. In mice, *Ezhip* -/- males are fertile, although H3K27me3 is increased in germ and sperm cells [32]. In females, EZHIP controls H3K27me3 deposition in growing oocytes and leads to reduced fertility [32]. Importantly, in addition to its likely role in stem and germ cells, EZHIP has been demonstrated to contribute to the development of ependymomas, which are neuroepithelial tumors of the central nervous system [34, 116, 117]. These tumor types are characterized by a reduction in H3K27me3 levels, often due to H3K27M mutations, that inhibit PRC2. EZHIP is often highly expressed in tumors that do not have an H3K27M substitution [119], suggesting that the cancer is either induced by an H3K27M mutation or high expression of EZHIP, which in both cases leads to reduced H3K27me3 levels [120]. EZHIP has been proposed to function as an oncohistone mimic in cancers [34]. This function may also be of relevance in other brain tumors where reduced H3K27me3 levels are critical [120–122]. Thus, EZHIP is a novel kind of PRC2-interacting protein that to date has only been identified in mammals and directly inhibits PRC2 enzymatic activity. It will be of interest to determine whether other, currently unidentified proteins may facilitate similar functions as EZHIP in regulating PRC2.

### Evolution of the chromatin binding of PRC2

**Fig. 5:**
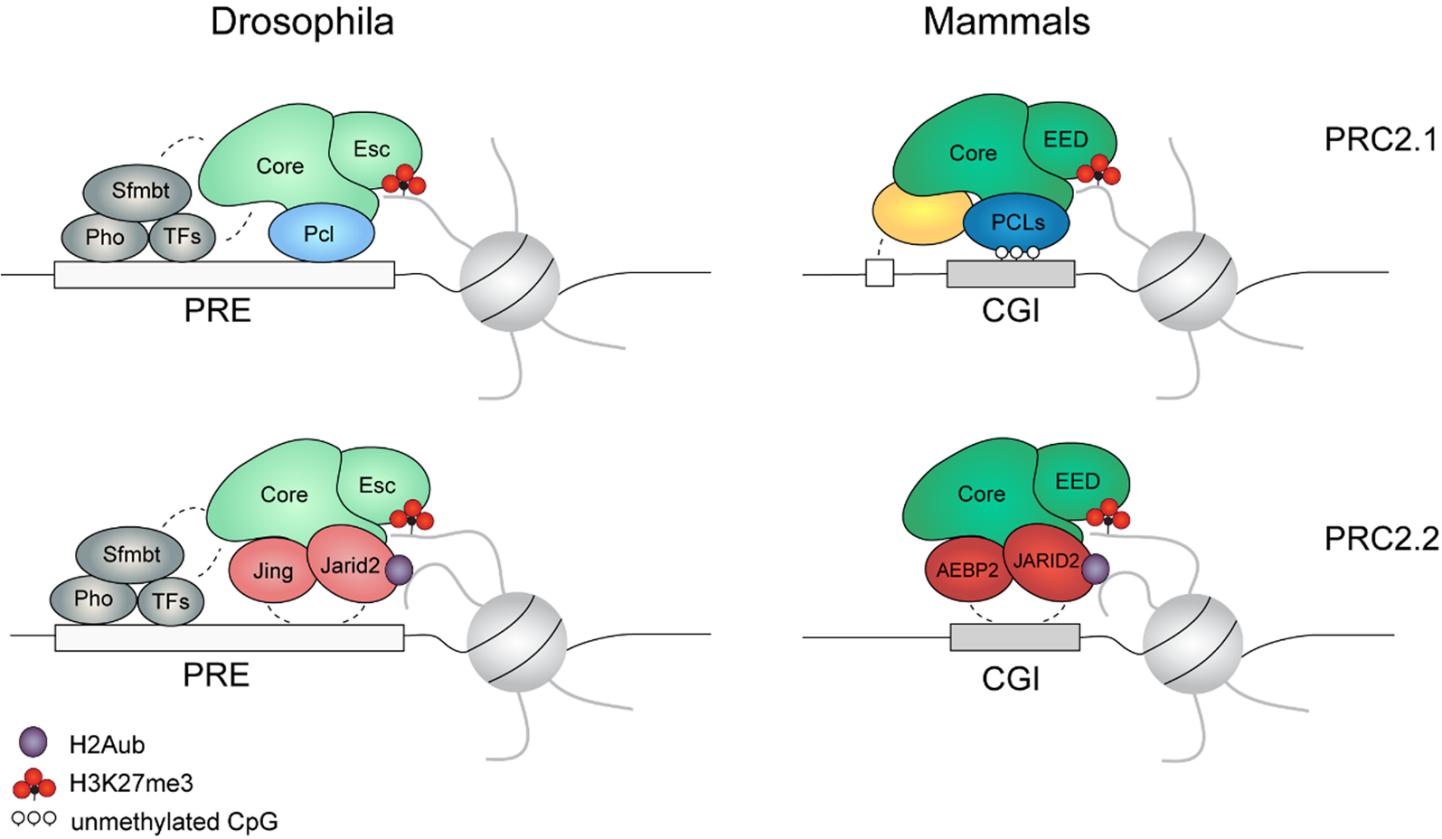
Simplified comparison of PRC2.1 and PRC2.2 chromatin binding in Drosophila and mammals. In Drosophila, PRC2 localizes to Polycomb response elements (PREs) and is recruited by Pho and Sfmbt proteins, as well as other transcription factors (TFs). Nonspecific DNA binding of Pcl, Jarid2 and Jing also contributes to PRC2 chromatin recruitment. In mammals, PRC2.1 is mainly located at unmethylated CpG islands (CGIs), recognized by the DNA binding winged-helix domain of the PCL proteins. Additional binding features of the associated factors may recruit distinct PRC2.1 subcomplexes to other DNA binding sites. PRC2.2 recruitment to chromatin depends on JARID2 recognition of H2Aub-modified nucleosomes, mediated by the PRC1 complex, as well as nonspecific DNA binding of AEBP2 and JARID2

During animal evolution, genome organization has altered significantly. Therefore, the mechanisms of PRC2 recruitment to its target locations have also changed. In Drosophila, PRC2 is mostly present at so-called Polycomb response elements (PREs), which are specific DNA elements in the Drosophila genome and are often found at the transcription start sites of developmental genes [123, 124] (Fig. 5). PREs are regions of several hundred base pairs that have, however, no specific consensus sequence. The zinc finger protein Pho and its homolog PhoL are essential for the recruitment of Polycomb proteins to many PREs in Drosophila [123]. These proteins are the only known Polycomb proteins with sequence-specific DNA binding activity [125]. Loss of Pho leads to the disruption of the recruitment of both PRC1 and PRC2. However, Pho does not directly interact with either PRC1 or PRC2 but forms a stable complex with Sfmbt [126], a noncanonical Polycomb protein (Fig. 5). How exactly Pho contributes to Polycomb recruitment in Drosophila remains uncertain but may include polymerization of the SAM domains of Sfmbt, Scm and Ph [126, 127]. Additionally, Pho is not sufficient for PcG targeting [128]. It is likely that the chromatin association of PRC2 (and PRC1) is enhanced by other DNA binding proteins [129, 130] or DNA binding features of other proteins in the complex. For example, the nonspecific DNA binding function of the Drosophila Pcl [75] or the ARID domain of Jarid2 [100] may be involved in the chromatin association of Drosophila PRC2. Other features, such as the ubiquitin-binding function of Jarid2, likely also contribute [11, 12, 105, 106] (Fig. 5).

The genome and chromatin organization in Drosophila and mammals differ significantly. In Drosophila, the genome is very compact with high gene density [131]. In mammals, the gene density is lower and in contrast to Drosophila, the DNA is widely methylated [132]. Furthermore, novel genomic features have evolved, such as the CpG islands, which are stretches of DNA rich in CpG dinucleotides [133]. They are key transcriptional regulatory elements often found at promoters, and are typically DNA unmethylated [133]. Early work showed that PRC2 often colocalizes with unmethylated CpG islands in mammals [84, 134, 135]. More recent work demonstrated that PRC2 directly associates with unmethylated CGIs via a specific DNA binding winged-helix domain of the Polycomb-like proteins [67, 83]. The PCL proteins are components of the PRC2.1 subcomplex, suggesting that the PCLs can only recruit a subset of PRC2 to CpG islands (Fig. 5). Indeed, deletion of all PCL proteins leads to a reduction but not a complete abolition of PRC2 recruitment [36, 37]. Thus, although CGIs are an important recruitment factor of PRC2, other features must play a role in this context, such as H2A-ubiquitin binding of JARID2 [105], or nucleosome binding of AEBP2 [106, 136] (Fig. 5). In addition to the function of various protein domains, PRC2 is also associated with RNAs, which may play critical roles in the recruitment process of PRC2 [137]. However, there are currently conflicting views as to whether RNAs are mainly involved in PRC2 recruitment [138], or whether they repel PRC2 from chromatin [139, 140], as extensively reviewed by Almeida et al. [137]. It also remains to be elucidated under which conditions an unmethylated CpG island does or does not recruit PRC2. A key factor appears to be transcriptional activity, which prevents the recruitment of PRC2 to actively transcribed genes [140, 141].

### Why has PRC2.1 diverged more during evolution than PRC2.2?

**Fig. 6:**
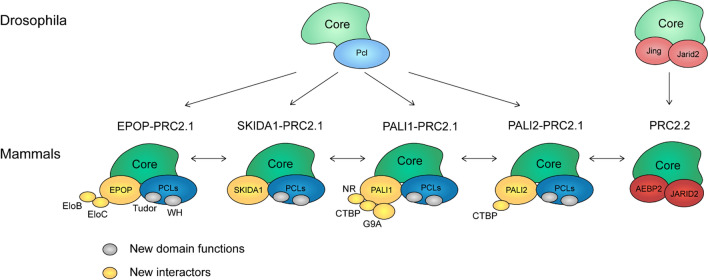
PRC2.1 diverged more strongly than PRC2.2, leading to several alternative PRC2.1 subcomplexes in mammals. Compared to Drosophila, these subcomplexes are characterized by novel domain features, such as specific DNA and histone binding functions (gray), as well as novel interacting partners (yellow)

Considering the above-described changes in the PRC2 subcomplexes and their members, it is evident that PRC2.1 has undergone more extensive changes than PRC2.2. Not only has the Pcl protein evolved into three alternative proteins, but novel components have also arisen (EPOP, SKIDA1, PALI1, PALI2), and several domains have developed new functions (Tudor and WH domains of the Pcl proteins). Thus, PRC2.1 exists as alternative complexes with distinct compositions and functions (Fig. 6). In contrast, PRC2.2 appears to have largely retained its configuration and function as far as they are currently understood. Why has PRC2.1 gained more novel functions compared to PRC2.2? One possible explanation is that Jing and Jarid2 occupy a large fraction of the nonvertebrate PRC2 core surface [95], while Pcl binds only a small fraction of Su(z)12 [25], keeping some parts of the PRC2 core accessible. This accessible surface may have been the starting point for the development of novel PRC2 binding sequences, such as those of SKIDA1/EPOP and PALI1/2. In addition, PRC2.1 has a high affinity for unmethylated CpG islands via the WH domain of the PCL proteins [67, 83]. Consequently, one could speculate that it was evolutionarily advantageous that PRC2.1 had additional regulatory features, allowing it to cope with the growing functions of CGIs [142]. For example, the interaction of EPOP with the transcription elongation factor Elongin BC may allow a mammalian-specific communication between the transcription machinery and PRC2 [22, 27], which could also involve Elongin A [118]. In contrast, the interaction of PALI1 with the H3K9 methyltransferase G9A could establish a link between heterochromatin regulation and Polycomb repression [21, 96] (Fig. 6).

In future research, it will be of interest to further understand the role of the various PRC2.1 complexes in distinct biological processes and their interplay with CpG islands, which may help to clarify why PRC2.1 shows greater evolutionary adaptation.

## Conclusion

The Polycomb repressive complex 2 shows major compositional and functional differences between invertebrates, such as Drosophila, and mammals. Continuous research on PRC2 has provided a comprehensive picture of the mechanistic roles of the PRC2 complex members, and the function of PRC2 in biological processes. Nonetheless, many open questions remain that are currently not fully answerable. Which determinants lead to the preferential establishment of the PRC2.1 or the PRC2.2 subcomplex, respectively? What are the roles of distinct PRC2.1 complexes, such as PALI1-PRC2.1 versus EPOP-PRC2.1, in gene regulation and during biological processes? Many PRC2 members, such as JARID2, EPOP, and PALI1 have extensive intrinsically disordered regions. Are these regions relevant for the function of PRC2? Are there further tissue-specific PRC2-interacting proteins, such as EZHIP, which have not yet been identified? Thus, despite remarkable progress, more work will be required to fully elucidate the function of distinct PRC2 subcomplexes at both the mechanistic and biological levels in Drosophila and mammals.

## Data Availability

Not applicable.

## Abbreviations

AEBP2: Adipocyte enhancer-binding protein 2
CRISPR: Clustered regularly interspaced short palindromic repeats
CGI: CpG island
CTBP: C-terminal binding protein
E(z): Enhancer of zeste
EZH2: Enhancer of zeste homolog 2
EED: Embryonic ectoderm development
EHMT1: Euchromatic histone-lysine N-methyltransferase 1
EPOP: Elongin BC and Polycomb repressive complex 2 associated protein
Esc: Extra sexcombs
EZHIP: EZH inhibitory protein
IP-MS: Immunoprecipitation-mass spectrometry
JARID2: Jumonji, AT rich interactive domain 2
MTF2: Metal-response element-binding transcription factor 2
NR: Nuclear receptor
PALI1: PRC2 associated LCOR isoform 1
Pcl: Polycomb-like
PHD: Plant homeodomain
PHF1: PHD finger protein 1
PHF19: PHD finger protein 19
PRC2: Polycomb repressive complex 2
PRE: Polycomb response elements
Sfmbt: Scm-like with four MBT domain-containing protein 1
SKIDA1: SKI/DACH domain-containing protein 1
Su(z)12: Suppressor 12 of zeste protein
WH: Winged-helix
